# A review of tumor treating fields (TTFields): advancements in clinical applications and mechanistic insights

**DOI:** 10.2478/raon-2023-0044

**Published:** 2023-09-04

**Authors:** Xing Li, Kaida Liu, Lidong Xing, Boris Rubinsky

**Affiliations:** College of Automation Engineering, Nanjing University of Aeronautics and Astronautics, Nan Jing, Jiang Su, China; Department of Mechanical Engineering, University of California Berkeley, Berkeley CA, United States of America

**Keywords:** tumor treating fields, clinical applications of TTFields, mechanisms of action of TTFields

## Abstract

**Background:**

Tumor Treating Fields (TTFields) is a non-invasive modality for cancer treatment that utilizes a specific sinusoidal electric field ranging from 100 kHz to 300 kHz, with an intensity of 1 V/cm to 3 V/cm. Its purpose is to inhibit cancer cell proliferation and induce cell death. Despite promising outcomes from clinical trials, TTFields have received FDA approval for the treatment of glioblastoma multiforme (GBM) and malignant pleural mesothelioma (MPM). Nevertheless, global acceptance of TTFields remains limited. To enhance its clinical application in other types of cancer and gain a better understanding of its mechanisms of action, this review aims to summarize the current research status by examining existing literature on TTFields’ clinical trials and mechanism studies.

**Conclusions:**

Through this comprehensive review, we seek to stimulate novel ideas and provide physicians, patients, and researchers with a better comprehension of the development of TTFields and its potential applications in cancer treatment.

## Introduction

Electromagnetic fields find various applications in medicine, including tissue ablation using thermal energy deposition at microwave and radiofrequency frequencies^1^, medical imaging with electrical impedance tomography^2^, nerve and muscle stimulation^3^, bone regeneration^3^, and more. Each of these applications employs specific electromagnetic field frequencies, intensities, and durations tailored to their purposes.

During the early 2000s, Professor Palti and his research group made an interesting discovery. They found that electric fields with low intensity (ranging from 1 V/cm to 3 V/cm, peak value) and intermediate frequency (between 100 kHz and 300 kHz) effectively inhibited the growth of tumor cells across various cell lines.^4,5^ This finding led to the development of a therapeutic modality known as Tumor Treating Fields (TTFields), which utilizes these specific electric field parameters to target and suppress tumor growth.^6,7,8^

TTFields have demonstrated their ability to inhibit tumor cell growth through both *in vitro* and *in vivo* studies. These fields are delivered to the tumor cells or solid tumor using insulated electrodes connected to an energy source, making the entire treatment protocol safe and noninvasive.^9^ For example, in the treatment of glioblastoma multiforme (GBM), a portable power supply located in the patient's backpack generates specific electric fields that are transmitted to the tumor through electrodes attached to the shaved scalp.^10^ The success of preclinical trials led to the approval of TTFields treatment by the Food and Drug Administration (FDA) for recurrent GBM in 2011, and newly diagnosed GBM in adult patients aged 22 years and older in 2015.^7,9,11^ These approvals were based on the significant effect of TTFields in prolonging the survival of GBM cancer patients. As a result, clinical trials have been conducted to assess the efficacy of TTFields treatment in other types of cancer as well. These include non-small cell lung cancer (NSCLC)^12,13,14,15^, platinum-resistant ovarian cancer (PROC)^16^, pancreatic adenocarcinoma (PAC)^17,18,19^, malignant pleural mesothelioma (MPM)^20,21,22^, and hepatocellular carcinoma (HCC).^23^ Additionally, clinical trials for TTFields treatment in other cancer types are currently ongoing.

Understanding the mechanism by which TTFields inhibit tumor cell growth is crucial for advancing the development of this promising technology. Previous research has suggested that TTFields exert mitotic inhibition effects on dividing cells through two main aspects. Firstly, the electric field force and torque disrupt the microtubule assembly process during prophase, leading to spindle damage.^6,19,24,25^ Secondly, during telophase, the inhomogeneous electric field in the cell generates dielectrophoresis (DEP) force^26,27^, driving free macromolecules and organelles towards the cleavage furrow, thereby unbalancing the intracellular microenvironment and ultimately causing the death of the dividing cell.^4,28,29^

However, while some physiological phenomena such as chromosome activity disorder^25,30^ or spindle disruption have been observed through fluorescence microscopy^31^, these alone cannot be considered direct evidence to support the above potential mechanisms. This is because these physiological phenomena may be related to biochemical imbalances rather than electric field mechanics. As a result, researchers are exploring the mechanism both theoretically^28,29^, and experimentally^4,25^ from the perspectives of biophysics and biochemistry.

This paper presents a comprehensive review of the current state of research on TTFields, focusing on the two most important aspects of this technology: clinical applications and anti-tumor mechanisms. By synthesizing the findings from a range of research works, literature, and reports, we aim to provide readers with a thorough understanding of the latest advancements in TTFields. Our review not only builds on previous research but also offers new insights that may inspire future directions for research and development. Ultimately, our goal is to contribute to the ongoing efforts to optimize the use of TTFields for cancer treatment.

## Clinical developments of TTFields

**FIGURE 1.**
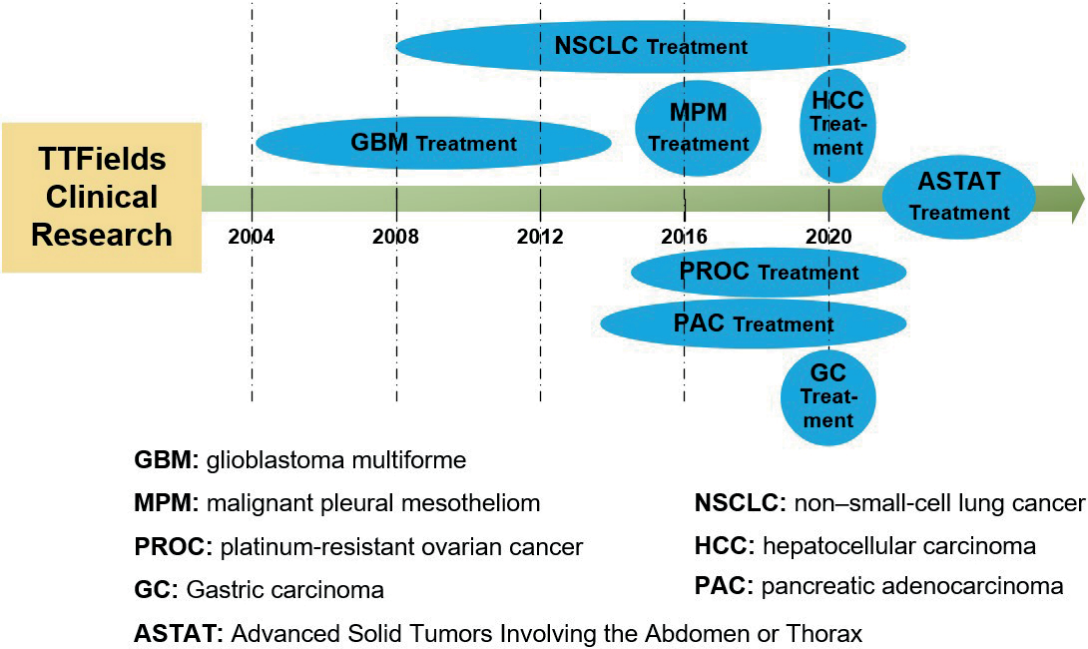
The process of TTFields clinical trials on typical tumor types.

Although TTFields have only been studied for less than two decades, numerous preclinical and clinical trials have been conducted to evaluate the efficacy of this therapy in treating various types of cancer. In Figure 1, we summarize the progress of TTFields clinical research on common tumor types. In the following subsections, we provide more detailed insights into the results of these studies.

### TTFields treatment on GBM

**FIGURE 2.**
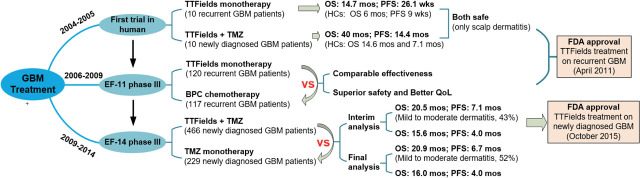
Clinical trials of TTFields treatment on glioblastoma multiforme (GBM) EF-11= controlled randomized phase III trial EF-11; EF-14 = phase III trial EF-14; OS = overall survival; PFS = progression-free survival; TMZ = temozolomide

GBM is the most common and aggressive form of brain tumor, has a survival rate of approximately 25% two years after diagnosis. Despite decades of research, few advances have been made in the treatment of this disease. The introduction of TTFields therapy provided a novel approach for the treatment of GBM. Clinical trials investigating the efficacy of TTFields therapy in GBM were initiated early on and are summarized as follows (Figure 2)

From 2004 to 2005, the first pilot trial was conducted to assess the safety and efficacy of TTFields therapy on GBM in humans. This trial consisted of two single arms, which involved 10 recurrent GBM patients (arm A) and 10 newly diagnosed GBM patients (arm B), respectively. In arm A, TTFields were used as the sole treatment following the failure of maintenance temozolomide (TMZ), while arm B received TTFields therapy combined with maintenance TMZ treatment.^32^ Further details regarding the TTFields setup and course plan can be found in.^32,33^ As this was a prospective pilot study, no related randomized control group was established. Therefore, the results were analyzed by comparing them to historical data.

The clinical trial yielded promising results, as evidenced by the comparison of outcomes in arm A and arm B to those of the historical controls (HCs). In arm A, patients treated with TTFields monotherapy achieved a median overall survival (OS) of 14.7 months and a median progression-free survival (PFS) of 26.1 weeks, compared to the HC group's respective outcomes of 6 months and 9 weeks.^34^ In arm B, which received TTFields combined with maintenance TMZ, had even more impressive outcomes, with a median OS and PFS exceeding 40 months and 14.4 months, respectively, compared to the HC group's median OS and PFS of 14.6 months and 7.1 months.^35^ In addition, no significant side effects, such as hematological, gastrointestinal toxicities, epileptic seizures, or cardiac arrhythmias, were observed in either arm A or arm B, except for contact dermatitis on the scalp.^33^ These results indicated TTFields technology is a safe and effective treatment option for GBM.

To promote the clinical advancement of TTFields, a controlled randomized phase III trial (EF-11) was conducted from 2006 to 2009, comparing the efficacy of TTFields monotherapy and best physician's choice (BPC) chemotherapy for recurrent GBM.^10^ The trial involved 237 patients, randomly assigned to receive either TTFields monotherapy (120 patients) or BPC chemotherapy (117 patients).^36^ Although the trial showed only comparable effectiveness between the two groups, TTFields monotherapy demonstrated superior safety and a better quality of life (QoL).

Based on the findings from the period spanning 2004 to 2009, TTFields therapy was granted FDA approval for the treatment of recurrent GBM on April 8, 2011.^11^

To further investigate the clinical application of TTFields for newly diagnosed GBM, an EF-14 phase III trial was conducted from 2009 to 2014, which enrolled about 700 patients. The patients were randomized 2:1 to receive either TTFields plus maintenance TMZ therapy (466 patients) or TMZ monotherapy (229 patients).^37^ According final endpoint analysis, the TTFields + TMZ group had a median PFS of 6.7 months and a median OS of 20.9 months, compared to 4.0 months and 16.0 months, respectively, in the TMZ monotherapy group.^38^ The only risk observed in TTFields + TMZ group is skin irritation beneath the electrodes (about 52% patients). Other common risks include headaches, insomnia and soft psychiatric symptoms were statistically non-significant. The significant improvement in PFS and OS by TTFields + TMZ treatment without obvious toxic side effects led to the second FDA approval of TTFields treatment on newly diagnosed GBM in October 2015.^11^ To date, TTFields treatment for GBM tumors has evolved into a relatively safe and patient-friendly therapy method.

### TTFields treatment on MPM and NSCLC

**FIGURE 3.**
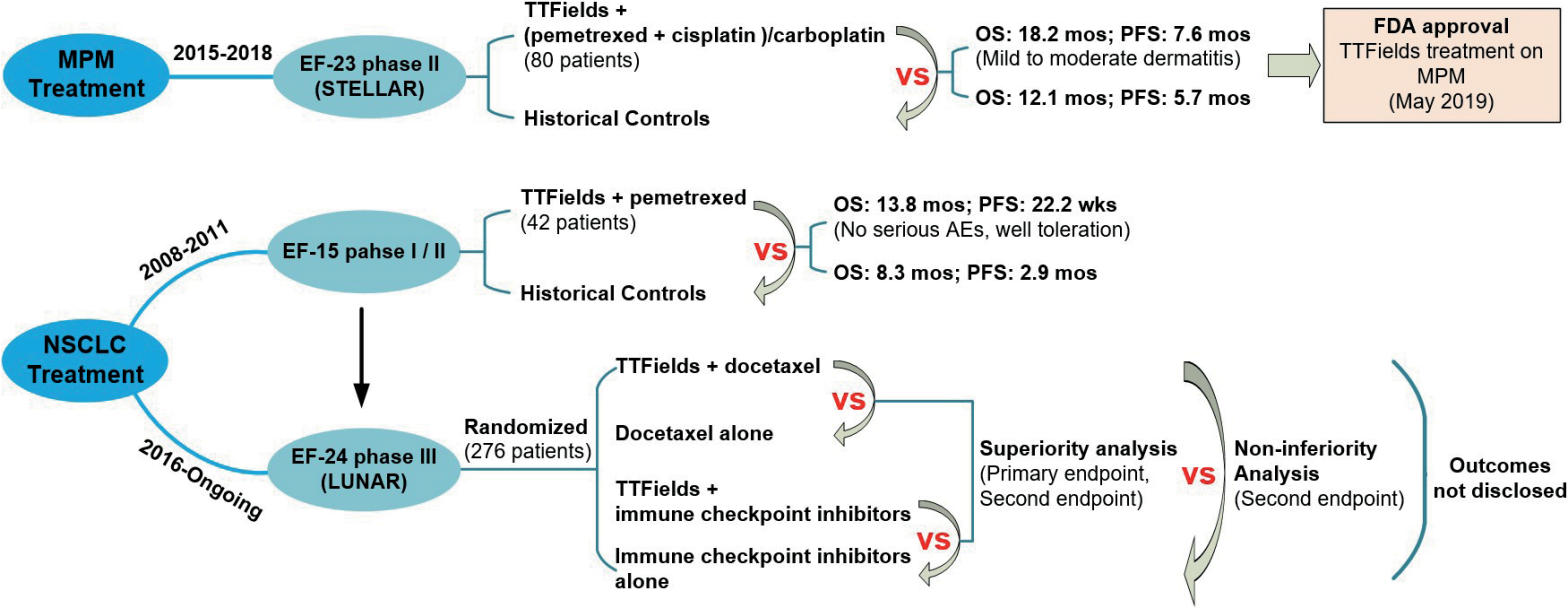
Clinical trials of TTFields treatment on malignant pleural mesothelioma and non-small cell lung cancer (NSCLC). EF-15 phase I/II = clinical trial NCT00749346; LUNAR = clinical trial NCT02973789; OS = overall survival; PFS = progression-free survival; STELLAR = clinical trial NCT 02397928

MPM has emerged as a leading cause of death, with incidence rates on the rise in Europe and Asia.^22^ Furthermore, the majority of MPM patients are diagnosed with diffuse disease and conventional therapies always have limited efficacy in such cases. In contrast, lung cancer is the primary cause of cancer-related mortality in the US, particularly among men, and NSCLC accounts for roughly 80% to 85% of all cases of lung cancer.^39^ To enhance therapeutic efficacy, researchers have postulated that TTFields could be a novel treatment modality for MPM and NSCLC, leading to the sponsorship of corresponding clinical trials. The developmental history can be succinctly summarized as follows (Figure 3).

Encouraged by the significant growth inhibition of mesothelioma cells *in vitro* treated by TTFields, the STELLAR trial (NCT 02397928) was conducted to evaluate the safety and efficacy of TTFields in combination with chemotherapy in MPM.^40^ This phase II clinical trial was a prospective, single arm study that involved 80 patients and was conducted from March 2015 to April 2018.^41^ The patients received standard doses of pemetrexed and cisplatin or carboplatin in combination with 150 kHz TTFields. With a minimum follow-up of 12 months, the median OS was 18.2 months compared to 12.1 months in the HCs, and median PFS was 7.6 compared to 5.7 months in HCs.^22,42^ Notably, the only toxic effects related to the treatment were mild to moderate dermatitis. The results indicated a meaningful improvement in MPM treatment with TTFields and standard chemotherapy. Although the STELLAR study has the limitations of single-arm design and the results need to be confirmed by a further randomized trial, however, the FDA approved TTFields therapy on MPM under the Humanitarian Device Exemption pathway, on May 23, 2019, was based on the meaningful clinical results.^22^

The previous phase III clinical trial of TTFields as monotherapy in GBM patients demonstrated its effectiveness and improvement of quality of life. Subsequently, an open-label EF-15 phase I/II clinical trial was conducted from May 2008 to September 2011 to treat NSCLC, which included 42 patients and was registered under the identifier NCT00749346.^43^ The preliminary phase I was to evaluate the adverse events (AEs) rate, while the second stage phase II continued to test feasibility and efficacy.^15^ Treatment in the trial was TTFields combined with pemetrexed. During the phase I trial, no serious AEs were reported and showed a well toleration, so the safety is confirmed. The statistical analysis of phase II results^15^ revealed that the median OS and median PFS of enrolled patients were 13.8 months and 22.2 weeks, respectively, compared to 8.3 months and 2.9 months in HCs reported by Hanna *et al*.^44^ This study suggested that TTFields could safely improve the disease control and treatment efficacy of NSCLC. Consequently, the followed EF-24 phase III clinical trial LUNAR (NCT02973789) was initiated in December 2016.^45^

The LUNAR study was designed as randomized to test whether the addition of TTFields to immune checkpoint inhibitors or docetaxel treatment can prolong the OS.^13^ This study includes a larger sample size of 276 patients and incorporates more comparative analysis. Three main comparative analysis will be reported: a) in primary endpoint, superiority analysis of OS between TTFields + docetaxel or immune checkpoint inhibitors vs docetaxel or immune checkpoint inhibitors alone; b) in secondary endpoint, superiority analysis of OS between TTFields + docetaxel vs docetaxel alone, and TTFields + immune checkpoint inhibitors vs immune checkpoint inhibitors alone; c) exploratory non-inferiority analysis of OS between TTFields + docetaxel vs immune checkpoint inhibitors alone. Additionally, in the second endpoint, PFS, QoL, etc. will be evaluated comprehensively. As the LUNAR study is still ongoing with an estimated completion date of September 2023, the outcome reports have not yet been disclosed.

### TTFields tretament on PROC, PAC and HCC

**FIGURE 4.**
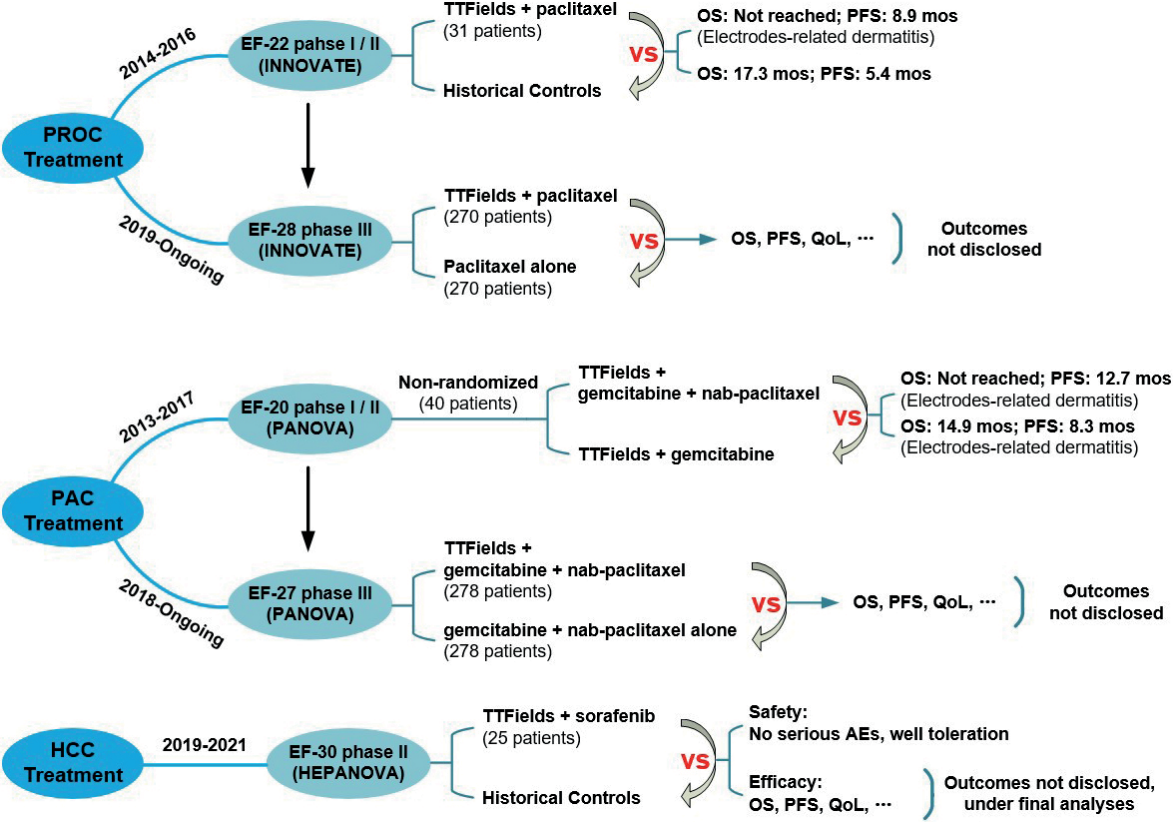
Clinical trials of TTFields treatment on platinum-resistant ovarian cancer (PROC), pancreatic adenocarcinoma (PAC) and hepatocellular carcinoma (HCC). AEs = adverse events; INNOVATE = a phase I/II clinical trial (EF-22, NCT02244502) and a phase III randomized controlled clinical trial (EF-28, NCT03940196); OS = overall survival; QoL = quality of life (QoL); PANOVA = a phase I/II clinical trial (EF-20, NCT01971281) and a larger randomized clinical phase III (EF-27, NCT03377491); PFS = progression-free survival

Previous studies have provided evidence that TTFields treatment is not associated with any serious adverse events. The mitotic inhibition mechanism of TTFields has also shown potential for use in the treatment of other types of cancers in the torso. Therefore, clinical trials on PROC, PAC and HCC were initiated. The reports are presented in Figure 4.

Ovarian cancer is a frequently occurring gynecological malignancy that is responsible for a high number of female fatalities. Chemotherapy remains the standard of care in advanced ovarian cancer patients. Due to the promising results of TTFields in many different types tumor treatment, several *in vitro* and *in vivo* experiments have been conducted to assess the feasibility of TTFields can be a novel approach to treat ovarian cancer.^46,47^ Furthermore, the clinical trials were also underway.^48,49^ Firstly, the INNOVATE trial (EF-22, NCT02244502), a phase I/II clinical trial was conducted from September 2014 to December 2016.^50^ This was a prospective, single arm, non-randomized pilot trial, designed to assess safety and preliminary efficacy of TTFields device used in PROC treatment. 31 patients were included in the INNOVATE study and treated by TTFields in combination with weekly paclitaxel, no control group was employed in this trial. The study results showed the median PFS of patients was increased to 8.9 months compared to 5.4 months (weekly paclitaxel alone) in HCs^16,51^, while the OS data was not reached in during the period, and the AEs found among patients were limited to electrodes-related dermatitis. Despite being a preliminary pilot trial, the INNOVATE study results showed promising response and survival data of PROC patients treated by TTFields. In addition to the INNOVATE trial, a phase III randomized controlled clinical trial (EF-28, NCT03940196) has been initiated to further investigate the safety and efficacy of TTFields in combination with weekly paclitaxel for PROC treatment was initiated in May 2019 and is currently ongoing (estimated completion in September 2023).^52^ The sample in this study consisted of 540 participants and were randomized assigned to two arms at a 1:1 ratio. Arm A received TTFields + weekly paclitaxel treatment compared to weekly paclitaxel treatment alone in arm B. The primary endpoints for this trial include OS, PFS, and QoL, and the results will be analyzed at the endpoint. However, as the study is ongoing, no results have been reported yet.^53^

Pancreatic adenocarcinoma is another lethal malignancy for which the standard of care is combination therapy with gemcitabine and nab-paclitaxel for advanced, unresectable patients.^54^ *In vitro* and *in vivo* studies have shown that TTFields can inhibit the growth of cancer cells and reduce the volume of pancreatic tumors.^19^ To assess the clinical efficacy and feasibility of applying TTFields to PAC therapy, corresponding clinical trials have been conducted. PANOVA (EF-20, NCT01971281) is the first clinical trial investigating the efficacy of TTFields in PAC treatment, which was conducted from November 2013 to December 2017.^55^ In this phase I/II trial, 40 patients were enrolled and non-randomly allocated into two arms. Treatments in the two arms were TTFields combined with weekly gemcitabine and TTFields in addition to gemcitabine plus nab-paclitaxel, respectively. Based on the study outcomes, the median OS and PFS of TTFields + gemcitabine group are 14.9 months and 8.3 months respectively. While TTFields + gemcitabine + nab-paclitaxel group had a PFS data of 12.7 months, but the OS was not reached at the end of follow-up period.^18^ Additionally, compared to the systemic chemotherapy alone, no increase in serious AEs except contact skin reaction. The phase I/II study demonstrated that TTFields + systemic chemotherapy is safe and well-tolerated in PAC advanced patients.

After the completion of phase I/II trial, a larger randomized phase III (EF-27, NCT03377491) with a sample size of 556 patients was initiated in May 2018 to further investigate the safety and efficacy of TTFields + chemotherapy vs chemotherapy alone.^56^ Therefore, in this trial, the experimental group received TTFields + gemcitabine + nab-paclitaxel treatment and the control group received gemcitabine + nab-paclitaxel alone. The study aims to analyze the results from multiple perspectives, including OS, PFS, QoL, toxicity profile and so on, but the results have not been reported yet as the trial is still ongoing and estimated to be completed in September 2024.

Liver cancer is another highly aggressive disease and is the third leading cause of cancer death globally.^57^ Unfortunately, 85% patients are diagnosed at advanced stage and their only option is chemotherapy. TTFields may be a potential treatment method based on its good performance *in vitro* and *in vivo* models.^58^ To assess the efficacy and safety of TTFields in combination with sorafenib to treat advanced HCC, a phase II clinical trial called HEPANOVA or EF-30 (NCT03606590) was conducted.^59^ This trial was a single arm, historical control experiment including 25 participants who were treated by TTFields + sorafenib form February 2019 to September 2021. According to the objective of the trial design, the outcomes would cover overall response rate, OS (or at 1 year), PFS (or at 6 and 12 months), AEs, and so on. Although the final results of the HEPANOVA trial are currently under final analyses^60^, there is a strong expectation that TTFields may emerge as a novel modality for HCC treatment.

### TTFields treatment on other advanced solid tumors involving the abdomen or thorax

Since receiving FDA approval as a treatment for recurrent GBM, TTFields has garnered significant attention as a promising physical therapy modality for various types of solid tumors, particularly those that are unresectable at advanced stages.^61^ Recently, a phase I clinical study (NCT05092373) has been initiated in April 2022 to evaluate the safety, AEs, and optimal dosage of TTFields therapy in combination with conventional chemotherapy, for advanced solid tumors located in the thorax or abdomen.^62^ This non-randomized study has recruited 36 participants diagnosed with various types of cancer, such as breast carcinoma, endometrial carcinoma, fallopian tube carcinoma, renal cell carcinoma, malignant abdominal neoplasm, and malignant thoracic neoplasm, among others. The study comprises two experimental arms without a control group, where the first arm receives TTFields + cabozantinib, and the second arm received TTFields + atezolizumab + nab-paclitaxel. The primary outcome will assess the safety and tolerability of TTFields and ulteriorly analyze the objective response rate, median OS and PFS in the secondary outcome. The outcomes of this trial will be made public after completion, which is estimated to be in September 2026.

In summary, since the initial clinical trial of TTFields treatment on recurrent GBM, several clinical studies have been conducted to evaluate the potential of TTFields as a new therapeutic approach for cancer treatment. While some ongoing trials have yet to report results, the current evidence is promising, and there is optimism regarding the efficacy of TTFields in cancer therapy.

## Progresses in revealing mechanisms of TTFields

As the development of science, researchers have an inherent curiosity to understand the underlying mechanisms that govern observed phenomena. In the case of TTFields, elucidating the mechanisms why TTFields have an inhibitory effect on cancer cells growth is an important research direction and many researchers involved in it.

**FIGURE 5.**
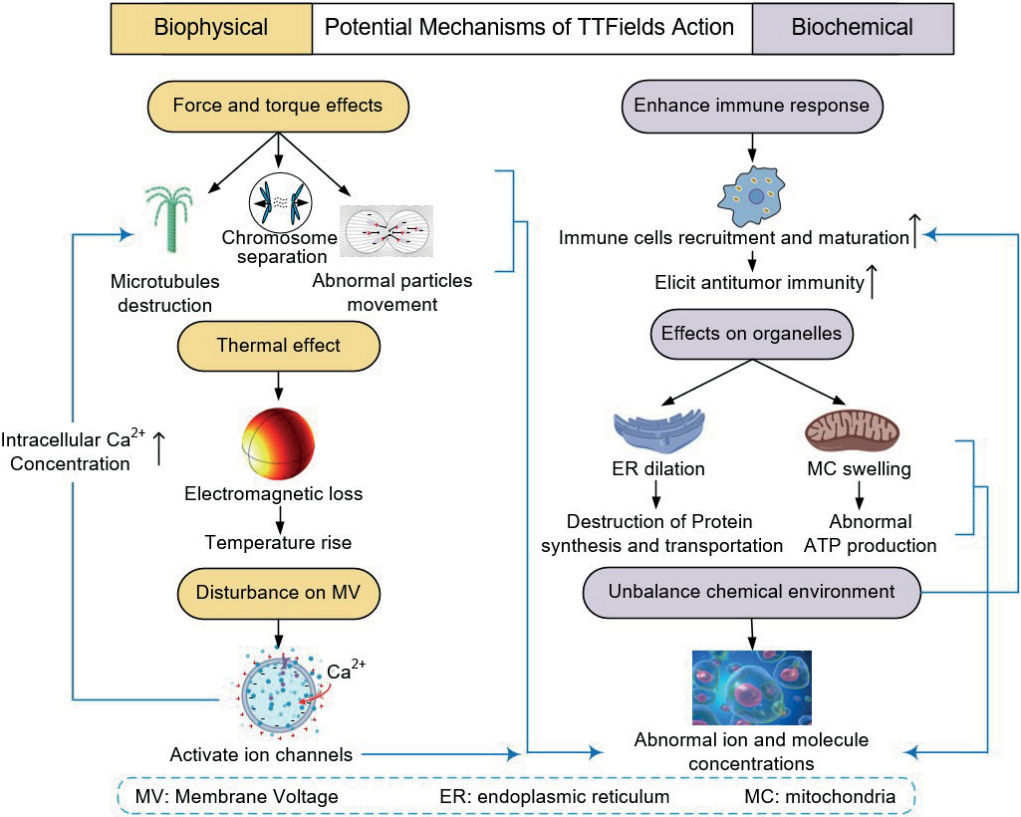
Researches on potential mechanisms of TTFields action.

Based on an overview of the existing studies, the mechanisms underlying the inhibitory effect of TTFields on cancer cell growth can be broadly categorized into two categories: biophysical and biochemical. The biophysical mechanisms pertain to the physical reactions between the electric field and cell or subcellular structures, encompassing electric field force, torque, dielectrophoresis (DEP) force, thermal effects, membrane voltage (MV), and related phenomena. While the biochemical mechanisms mainly investigate whether TTFields interfere with intracellular and extracellular chemical environments or even intercellular communication. It should be noted that these mechanisms are often interconnected and there is no rigid boundary between them. A schematic illustration of the interplay between biophysical and biochemical mechanisms is presented in Figure 5. In this review, we will provide a detailed exploration of the mechanisms involved in both categories.

### Force and torque effects on subcellular structures

Intracellular electric particles and subcellular structures are abundant in cells. When exposed to external electric fields, the resulting forces and torques can exert a range of effects on these subcellular structures, influencing their activity and morphology.

One widely accepted hypothesis for the inhibitory effect of TTFields on cancer cell growth is the cytoskeleton disruption theory. According to this theory, the electric field force and torque generated by TTFields can destroy the cytoskeleton and interfere with the cell division process, ultimately leading to cell death. This hypothesis is supported by several observations. Firstly, tubulin, the basic unit of microtubules, is a highly charged dimer protein with an electric dipole moment.^63,64^ When subjected to an external electric field, the geometrical orientation of tubulin dimers is twisted by electric field torque^4,65^ making it difficult for them to polymerize together, and resulting in cytoskeleton destruction. The cytoskeleton plays a crucial role in mitotic processes and maintaining proper cell shape, such as spindle formation, chromosomes traction and arrangement, and serving as a bridge for motor proteins. Therefore, cytoskeleton disruption can cause not only mitotic catastrophe, but also morphological abnormalities. This microtubule damage mechanism, initially proposed by the discoverer of TTFields, provides a plausible explanation for the observed antitumor effects of TTFields.

Although some experimental phenomena including abnormal spindle structure^19,24^, chromosome aneuploidy^25,66^, nuclear dysmorphologies^31,67^, are observed *in vitro* in different cell lines treated by TTFields, skepticism and even contrary conclusions persist.^28,29,68,69^ In fact, the proposed mechanism of cytoskeleton destruction caused by electric field force and torque has been challenged by a logical problem. It seems reasonable that the experimental results proved the above assumption, however it is possible that microtubule damage is not directly caused by force or torque, but rather by other indirect causes. In^29^ researchers have attempted to address this issue by modeling single cells and intracellular substructures, and calculating electric field force and torque on the tubulin dimer or chromosome traction theoretically based on detailed electric parameters.^70,71,72,73^ According to the computation results, they drew the conclusion that: a) the torque on the dimer imposed by TTFields is several orders smaller than random Brownian thermal motion energy; b) the electric field force between the microtubule terminal and kinetochore generated by TTFields is also much weaker than the natural electrostatic attraction. Therefore, the results suggesting that more rigorous scientific methods and more precise instruments are needed to further study this mechanical effects of TTFields.

### Dielectrophoresis effects during mitotic telophase

In the presence of a uniform electric field, electric polar particles maintain a balance of electric field forces. However, in non-uniform electric fields, they tend to undergo dielectrophoresis (DEP) effect^27^, which causes their movement. The DEP force is primarily dependent on factors such as the electric field gradient, particle size, and permittivity.^74^ Biological cells contain numerous polar particles such as proteins and organelles, which can be influenced by the DEP effect when exposed to external electric fields. During the later stage of mitosis, two daughter cells will be connected by the cleavage furrow, where is very narrow with great electric field gradient. Therefore, the DEP force is much stronger in the cleavage furrow. Pushed by the DEP force, macromolecules and some free organelles will move towards the cleavage furrow, consequently, impaired cell division occurred or unhealthy daughter cells are born.^75^

It is important to highlight that the orientation of the cell division axis is a significant factor affecting the intensity of the electric fields in the cell. When the axis is aligned parallel to the external electric field, a larger number of electric field lines are concentrated in the cleavage furrow, resulting in more significant DEP effects.^75^ Additionally, the duration of the telophase stage also plays a crucial role in determining the interference effect of DEP on cell division, as the velocity of particle movement triggered by the DEP force is slow due to the viscous cytoplasm.^76^ Theoretical analysis in^29^ has further examined this point. The effects of cell division axis orientation and duration of the telophase stage may explain why only a subset of cells is inhibited, rather than all. Briefly, despite the DEP effect generated by TTFields should also be further confirmed, it seems to be one of the more likely mechanisms.

### Thermal effect caused by electromagnetic loss

The application of electromagnetic loss thermal effect has been successfully employed in clinical treatments, such as radiofrequency ablation and microwave ablation. TTFields are low-intensity and intermediate-frequency, intuitively, the thermal effect could not be significant. To clarify this matter, Li *et al*. simulated the electromagnetic power dissipation distribution and temperature rise in the single cell.^77^ Additionally, infrared camera was also employed to capture the temperature change. Expectedly, the results showed no significant temperature rise in both simulation and experiment, which suggests that TTFields may not generate a significant thermal effect. Berkelmann *et al.*^78^ conducted a study to investigate the specific absorption rate (SAR), which is the standard measure to determine the safe exposure limits to electromagnetic fields. They measured the steady temperature in the cell dishes exposed to electric fields with different intensities. The results showed that only slight temperature increased (under 0.2 K) in the dish center. Moreover, in several animal experiments and clinical treatments, only a mild increase in skin temperature was monitored.^19^ To further improve safety, clinical treatment devices have been designed with temperature sensors located under the electrodes. These sensors are able to detect when the temperature exceeds 41°C, at which point the power is automatically lowered.^36^ Based on the combination of theoretical and experimental results, to our best knowledge, it is generally agreed upon that thermal injury can be excluded as a potential mechanism for the effects of TTFields.

### Disturbance of cell membrane voltage

As the barrier between inside and outside the cell, cell membrane plays a pivotal role in maintaining the intracellular environment and keeping external interference at bay. Cell membrane possess certain voltage, which is crucial for ensuring normal ionic concentrations and performing other vital physiological functions. When the cell is exposed to an external electric field, an induced voltage will be superimposed on the natural cell membrane voltage (MV). Once the disturbance exceeds the tolerance of normal MV, the permeability of cell membrane will be affected, for example, the well-known electroporation.^79^

Whether TTFields will change the permeability of cell membrane has aroused researchers’ attention, interestingly, some positive evidences has emerged in recent studies. Specifically, in a theoretical analysis conducted by Li *et al*,^29^ authors calculated the TTFields induced voltage on the cancer and normal single cell membrane, and found cancer cell membranes were affected to a greater extent than healthy cell membranes. This led the authors hypothesized that TTFields can specifically increase the permeability of cancer cell membrane, particularly by impacting the function of ion channels. Moreover, experimental findings by Chang *et al*.^80^ revealed that TTFields can increase the permeability of GBM cells and induce the formation of reversible pores in the cell membrane, as observed through scanning electron microscopy.

To investigate the effect of TTFields on cell membrane ion channels, Neuhaus *et al*. utilized the patch-clamp technique to record the potential change of cell membrane potential and their results indicated that TTFields activate K^+^ and Ca^2+^ ion channels on the cell membrane.^81^ Disruption on the cell membrane permeability may offer a reasonable explanation for the observed improvement in therapeutic efficacy when TTFields are combined with chemotherapy. Moreover, abnormal ion channel function can cause electrolyte imbalances in cells, thereby affecting the formation and activity of subcellular structures. For instance, the concentration of Ca^2+^ and Mg^2+^ have been found to be an essential factor affecting microtubule assembly.^29,82^ This founding may explain the disorder of microtubule polymerization caused by TTFields via disturbing the cell membrane permeability, but not by the direct mechanical torque on the tubulin. Although experimental evidence suggests that TTFields can increase cell membrane permeability, the relationship between TTFields frequency, pore size, and ion channel opening remains unclear and requires further investigation.

### Effects on immune response

The immune system is much important for human to resist diseases. It is a common therapy method to treat diseases by stimulate the immune system and improve the immune ability with drugs or other physical means, such as cancer immunotherapy. Exploring whether TTFields activate specific immune responses to arrest tumor cell growth is an area of potential significance.

Preliminary evidence suggests that this may be the case. For example, in^83^, the authors demonstrated TTFields can promote immune cells recruitment and maturation, resulting in eliciting antitumor immunity. Furthermore, they also showed the combination of TTFields with anti-PD-1 therapy resulted in a significant improvement in the antitumor effect.^83,84^ Similarly, Chen *et al*.^85^ reported that TTFields can be a unique activator of STING and AIM2 inflammasomes to improve antitumor immunity. This special mechanism may be generalizable and could be further explored a new avenue for antitumor immunity in other tumors. Although some preliminary findings have shown the effect of TTFields on immune responses, there is still a paucity of related studies. Further research is needed to confirm and investigate how TTFields stimulate and interact with immunity in more tumor models.

### Effects on organelles’ activities and morphology

Cells, the smallest units that make up most of life, are highly complex. Their normal physiological activities depend on the proper function of various organelles. Examining the mechanisms of TTFields action from the perspective of organelles may reveal unexpected findings.

Early research suggested that DEP force may be responsible for moving free organelles towards the cleavage furrow during the mitotic telophase. However, the impact of TTFields on the activities and morphology of organelles has not yet been thoroughly investigated. In recent years, some researchers have found that TTFields can trigger an increase in intracellular phagolysosome formation both *in vitro* and *in vivo* models, they believed this phenomenon may be a potential mechanism related to the cell death caused by TTFields.^86,87,88^ endoplasmatic reticulum (ER) is a critical organelle involved in protein synthesis and transportation. In^83,86^ the authors demonstrated they have observed abnormal morphology of ER when cells are exposed to TTFields, however, the precise relationship between TTFields and ER dysfunction in the context of induced cell death has yet to be fully elucidated. All biological activities of cells are inseparable from energy, as the energy unit, ATP is produced by a meritorious organelle called mitochondria (MC). When cancer cells are exposed TTFields, not only the direct morphological swelling change of MC was observed, but also abnormal ATP concentration was found out of the cell^83,89^, which could be related to protein production disruption and cell apoptosis. Due to the fact that the tumor cells are much more aggressive to divide than healthy cells, they are more reliant on ATP energy generated by MC. Therefore, the disruption on MC structure and function by TTFields can be most likely mechanism to selectively inhibit cancer cells growth but with minimal effect on normal cells.

We believe that TTFields may affect other organelles beyond those discussed above, but the relationship between the observed experimental phenomena and the underlying mechanisms requires further clarification. Additionally, more rigorous logical analyses are needed to fully understand the effects of TTFields on organelles.

## Conclusions

TTFields therapy is a remarkable discovery that employs physical means to treat cancer, offering unique advantages that have led to its FDA approvals for treating GBM and MPM, with other related approvals pending. Promising results of clinical trial investigating the TTFields therapy in GBM treatment have prompted the launch of numerous clinical trials exploring its potential in the treatment of thoracic and abdominal cancers, both with and without traditional chemotherapy. Although not all experimental data are fully disclosed, published results have revealed significant therapeutic effect enhancement and low adverse events associated with TTFields therapy. Even for trial results that have yet to be released, researchers remain confident in achieving positive outcomes. Meanwhile, the mechanisms behind the effects of TTFields therapy have received increasing attention, moving from observational studies to understanding the underlying scientific principles, this is a scientific logic from what to why. Two most popular perspectives of the mechanisms are the cytoskeleton destruction caused by electric field force and DEP effect on subcellular structures. Besides, the mechanism studies also focus on TTFields effects on cell membrane voltage, immune response, and organelles. While some corresponding experimental phenomena have been observed *in vitro* or *in vivo*, the internal relationship between the phenomena and theory should be clarified based on more rigorous logic. Furthermore, it is plausible that the mechanism of TTFields therapy is not singular but rather a combination of multiple reasons.

To summarize, TTFields cancer treatment is a relatively novel technique that requires further development. In this paper, we reviewed two important aspects of TTFields: the clinical development and progresses in mechanism study. Many clinical trials were initiated to test the efficacy and safety of TTFields treatment, and are currently ongoing. The promising results of these studies suggest a bright future for TTFields as a cancer treatment. Nevertheless, the mechanisms of action of TTFields are still not fully revealed. Future research should focus on elucidating these mechanisms to optimize the therapeutic effect of TTFields. This can be achieved through a better understanding of the scientific mechanisms behind TTFields, and enhance its therapeutic effect through optimal combinations with traditional therapy means.
